# Socioeconomic Status and Longitudinal Lung Function of Healthy Mexican Children

**DOI:** 10.1371/journal.pone.0136935

**Published:** 2015-09-17

**Authors:** David Martínez-Briseño, Rosario Fernández-Plata, Laura Gochicoa-Rangel, Luis Torre-Bouscoulet, Rosalba Rojas-Martínez, Laura Mendoza-Alvarado, Cecilia García-Sancho, Rogelio Pérez-Padilla

**Affiliations:** 1 Epidemiology and Social Science in Health, National Institute of Respiratory Diseases (INER), Mexico City, Mexico; 2 Population Health Research Center, National Institute of Public Health (INSP), Mexico City, Mexico; Johns Hopkins University, UNITED STATES

## Abstract

**Introduction:**

Our aim was to estimate the longitudinal effect of Socioeconomic status (SES) on lung function growth of Mexican children and adolescents.

**Materials and Methods:**

A cohort of Mexican children in third grade of primary school was followed with spirometry twice a year for 6 years through secondary school. Multilevel mixed-effects lineal models were fitted for the spirometric variables of 2,641 respiratory-healthy Mexican children. Monthly family income (in 2002 U.S. dollars [USD]) and parents’ years completed at school were used as proxies of SES.

**Results:**

Individuals with higher SES tended to have greater height for age, and smaller sitting height/standing height and crude lung function. For each 1-year increase of parents’ schooling, Forced expiratory volume in 1 sec (FEV_1_) and Forced vital capacity (FVC) increased 8.5 (0.4%) and 10.6 mL (0.4%), respectively (*p* <0.05) when models were adjusted for gender. Impact of education on lung function was reduced drastically or abolished on adjusting by anthropometric variables and ozone.

**Conclusions:**

Higher parental schooling and higher monthly family income were associated with higher lung function in healthy Mexican children, with the majority of the effect likely due to the increase in height-for-age.

## Introduction

Lung function, an important component of the evaluation of children with respiratory problems, is influenced by gender, height, and age, but also by prenatal exposures, genetic factors, ethnicity, obesity, altitude of residence, tobacco smoking, air pollution, nutrition, Socioeconomic status (SES), and lung disease [1]. Data from a variety of studies consistently show that low SES is associated with lower mean levels of pulmonary function [2–5]. However, data of children from developing countries is scarce, especially if studied in longitudinal fashion [6]. The pattern of increase in lung function may differ if obtained from longitudinal or cross-sectional studies [7] because, in the latter, the effects of age on lung function are confounded by secular time and by the presence of the multiple birth cohorts assembled in a cross-sectional study. A longitudinal study may better describe the growth spurt in adolescents [8] and the time sequence of events, such as the impact of general state of health and nutrition and SES on lung growth. The main objective of the present study was to evaluate the effect of SES on the lung function of healthy Mexican children from a cohort assembled in Mexico City.

## Materials and Methods

### Population and study design

The Metropolitan Study to Evaluate the Chronic Effects of Pollution in School-age Children (EMPECE) was undertaken in Mexico City beginning on April 23, 1996, with children in third grade of primary school [9]. The protocol was approved by the Ethics Committee of the Mexican National Institute of Respiratory Diseases (INER). All parents provided written informed consent for the study subjects.

We selected 10 fixed-site air-monitoring stations in Mexico City and randomly selected 39 elementary schools from among those located within a 2-km radius of the stations. The study cohort consisted of students at the selected schools who were 8 years of age at the beginning of the study, who had not been diagnosed as having asthma, and whose parents had signed a letter of informed consent. A substantial number of children entered or left the cohort during the course of the study. At baseline, a questionnaire was completed by the parents of 1,819 children, and a spirometric test was administered to each child (phase 1). The following evaluations occurred every 6 months during the spring and autumn seasons of each year until the end of the children’s primary school education in 1999. Children remaining in the same schools studied during secondary school were followed for 3 additional years until 2002; thus, we collected information for a total of 12 evaluations [9–11].

### Spirometry testing

Spirometry tests were conducted using identical computerized dry-rolling seal spirometers (922 Spirometer; SensorMedics, USA) that were calibrated each morning prior to data collection with a 3-L syringe (SensorMedics). We recorded only the expiratory part of Forced expiratory maneuvers (FEM) and analyzed Forced expiratory volume in 1 sec (FEV_1_), Forced vital capacity (FVC), and their ratio (FEV_1_/FVC). Tests were performed at the school during morning and early afternoon hours. As many as eight FEM were conducted for each child to obtain three acceptable ones according to 1994 American Thoracic Society (ATS) criteria [12]. Additional details on the spirometry methodology, including sustained quality control along the study, were described in a previous report [10].

### Respiratory-healthy children

Children with self-report of asthma, tobacco smoking, chronic respiratory symptoms (cough, wheezing, phlegm, dyspnea), or children in the >95% percentile of Body mass index (BMI) for age according to growth charts from the Centers for Disease Control and Prevention (CDC) [13], were excluded from the analysis because those conditions would tend to reduce lung function and confound a relationship with SES. Children <8 years of age were scarce and were also excluded, as performance of spirometry is less reliable than in older children. Although some of these diseases, symptoms, and exposures could also be associated with poverty, we preferred to analyze the impact of SES on lung function in a healthy population from the respiratory point of view, but also included statistical models with data from the whole cohort.

### Socioeconomic status

Socioeconomic status was based on monthly family income (in 2002, log-transformed U.S. dollars [USD]) assessed at each evaluation and by parents’ schooling (years completed at school), both reported by the parents and categorized in tertiles for analysis. Parents’ schooling was built taking the average of the highest completed school years of both parents. Income changed over time, while parents’ schooling was constant.

### Ozone measurement

The impact of several air pollutants on the lung function of the cohort was previously reported (9). As an indicator of air pollution, we included ambient O_3_ from 10 government air-monitoring stations, assigning for each child data from the station closest to their school. We calculated 8-h ozone means (parts per billion [ppb] between 10 a.m. and 6 p.m.), whereas long-term exposure for each day of the study period was estimated as the average over the previous 6 months of the daily O_3_ 8-h mean [9].

### Statistical analysis

We estimated tertiles of schooling and compared spirometric and anthropometric variables by Analysis of variance (ANOVA). We then fitted multilevel mixed-effects linear models adjusted for age and gender to determine the association of SES (monthly family income or parents’ years at school) with spirometric variables (log-transformed FEV_1_ and FVC) along time with and without height, ozone (average over the previous 6 months of the daily O_3_ 8-h mean, as a general indicator of air pollution), interaction between SES variables and ozone and secondhand smoke (the presence of any person smoking inside the home). We also fitted multilevel mixed-effects linear models for spirometric variables expressed as Z-score [14] including SES variables, second hand smoke and ozone. Sitting height was measured only during the 4th evaluation; therefore, we could include sitting height/standing height as additional confounder and also as a consequence of SES only for observations during this phase. We also estimated the height-for-age Z-score [13] and the sitting height/standing height ratio (only available in the 4th evaluation) in order to compare groups (by tertiles), and we established a relationship between these two variables with spirometric and other anthropometric measurements.

The analysis was conducted using the software STATA v.13 software (Stata Corp., College Station, TX, USA).

## Results

Of the 3,177 children finally included in the cohort, 536 presented at least one of the exclusion criteria as follows: 28 were <8 years of age; 67 reported having asthma; 190 were smokers, and 251 had obesity. Thus, we collected a total of 14,165 measurements from 2,641 children who were considered respiratory-healthy and who were 8–17 years of age. Mean age at study inclusion was 9.2 years of age (Standard deviation [SD], 1.1 years) for girls and 9.5 years (SD, 1.2 years) for boys. Observations per individual ranged in number from 1‒12 (median, 4 observations, and interquartile range, 4 observations). Mean follow-up duration was 2.5 years (SD, 1.9 years). Study participants’ characteristics by follow-up phase can be observed in (S1 and S2 Tables) [14].

Tables 1 and 2 describe socioeconomic variables and ozone exposure by follow-up phase and gender. Response rate (RR) of the monthly family income variable was 84.5% along the follow-up period. Table 3 depicts the characteristics of the study population in phase 4, the only phase including measurements of sitting height/standing height. Table 4 presents results by tertiles of parents’ schooling, while S3 Table separates data by tertiles of monthly family income. The lowest tertile had a reported mean monthly family income of $119.4 USD and 7.7 years at school, the middle tertile, $360.6 USD and 11.8 years at school, and the highest tertile, $949.3 USD and 16.0 years at school.

**Table 1:** Socioeconomics status (SES) and ozone exposure of the boys studied (means and Standard deviation [SD]).

	* *	Monthly income*	Parents’ schooling	O_3_ **
Phase	N	(U.S. dollars) ($)	(years)	(ppb)
1	676	612.0 (522.4)	11.5 (3.7)	79.9 (11.3)
2	718	556.4 (499.4)	11.3 (3.7)	64.0 (13.3)
3	853	481.8 (426.3)	11.4 (3.7)	70.1 (9.1)
4	776	479.4 (420.0)	11.4 (3.7)	64.2 (10.6)
5	657	420.4 (364.5)	11.4 (3.8)	75.2 (8.4)
6	808	416.1 (363.3)	11.4 (3.8)	65.2 (10.3)
7	812	369.9 (319.7)	11.5 (3.7)	73.6 (10.1)
8	322	436.0 (319.2)	12.7 (3.5)	69.9 (8.2)
9	282	449.1 (308.8)	12.5 (3.5)	58.9 (9.0)
10	280	468.4 (298.0)	12.5 (3.6)	65.2 (7.3)
11	231	453.7 (298.3)	12.7 (3.5)	54.2 (8.0)
12	240	493.9 (291.9)	12.6 (3.5)	59.0 (6.7)

*2002 U.S. dollars (USD): Exchange rate $9.66 Mexican pesos per USD; ppb = parts per billion

** Previous 6 months of the daily O_3_ 8-hour mean (parts per billion [ppb] from 10 a.m. to 6 p.m.).

**Table 2:** Socioeconomics status (SES) and ozone exposure of the girls studied (means and Standard deviation [SD]).

	* *	Monthly income*	Parents’ schooling	O_3_ **
Phase	N	(U.S. dollars) ($)	(years)	(ppb)
1	776	566.3 (518.5)	11.3 (3.5)	78.4 (10.3)
2	795	537.4 (489.5)	11.2 (3.2)	64.2 (12.4)
3	949	468.6 (422.0)	11.3 (3.5)	69.8 (8.3)
4	895	471.5 (423.3)	11.3 (3.5)	63.8 (9.7)
5	748	426.0 (373.7)	11.4 (3.6)	74.3 (8.4)
6	892	404.6 (363.0)	11.4 (3.5)	64.4 (9.9)
7	912	372.6 (318.5)	11.4 (3.5)	72.8 (9.5)
8	351	393.2 (295.0)	12.0 (3.4)	68.8 (6.8)
9	327	398.4 (284.7)	12.0 (3.4)	57.9 (7.8)
10	317	392.9 (283.1)	12.0 (3.5)	64.6 (6.8)
11	269	414.8 (270.8)	12.0 (3.5)	53.5 (7.6)
12	279	396.9 (248.6)	12.0 (3.5)	58.3 (5.3)

*2002 U.S. dollars (USD): Exchange rate, $9.66 Mexican pesos per USD

**Previous 6 months of the daily O_3_ 8-hour mean (parts per billion [ppb] from 10 a.m. to 6 p.m.).

**Table 3:** Characteristics of the studied population in phase 4 of the study. SD = Standard deviation; BMI = Body mass index; FEV_1_ = Forced expiratory volume at 1 sec; FVC = Forced vital capacity; FEV_1_/FVC = ratio of FEV_1_ and FVC.

Variable	Mean (SD)
Boys [*n* (%)]	776 (46.4)
Age (years)	10.7 (0.8)
Weight (kg)	34.8 (6.9)
Standing height (cm)	139.5 (7.3)
Sitting height (cm)	73.8 (4.4)
Sitting height/Standing height (%)	53.0 (2.2)
BMI (kg/m^2^)	17.8 (2.4)
FEV_1_ (L)	2.15 (0.37)
FVC (L)	2.41 (0.41)
FEV_1_/FVC (%)	89.4 (5.5)
BMI-for-age (Z-score)	0.0 (1.0)
Height-for age (Z-score)	-0.4 (1.0)
Weight-for-age (Z-score)	-0.2 (1.0)
Monthly income* ($USD)	475.1 (421.7)
Highest schooling	11.0 (3.6)
O_3_ ** ppb	64.0 (10.1)

*2002 U.S. dollars (USD): Exchange rate, $9.66 Mexican pesos per 1 USD

**Previous 6 months of the daily O_3_ 8-hour mean (parts per billion [ppb] from 10 a.m. to 6 p.m.).

**Table 4:** Characteristics of the children studied by tertile of parents’ schooling.

Variable	1 (min 3, max 9)	2 (min 11, max 12)	3 (min 14, max 18)	*P* value
FEV_1_ (L) [mean (SD)]	2.35 (0.66)	2.42 (0.70)	2.45 (0.69)	< 0.001
FVC (L) [means (SD)]	2.63 (0.71)	2.71 (0.75)	2.75 (0.76)	< 0.001
FEV_1_/FVC (%) [mean (SD)]	89.4 (6.1)	89.2 (6.1)	89.0 (6.2)	< 0.001
Age (years [mean (SD)]	11.4 (1.7)	11.4 (1.8)	11.4 (1.8)	< 0.001
Standing height (cm) [mean (SD)]	142 (11.0)	144 (11.4)	145 (11.3)	< 0.001
Sitting height/Standing height (%) [mean (SD)]*	52.8 (2.3)	52.7 (2.3)	52.3 (2.3)	0.0043
Weight (kg) [mean (SD)]	37.2 (10.1)	38.5 (10.4)	39.3 (10.5)	< 0.001
BMI (kg/m^2^) [mean (SD)]	18.2 (2.8)	18.3 (2.8)	18.5 (2.9)	< 0.001
BMI-for-age (Z-score)	0.01 (1.08)	0.06 (1.22)	0.09 (1.17)	< 0.001
Height-for-age (Z-score)	-0.67 (0.96)	-0.43 (0.91)	-0.32 (0.88)	< 0.001
Weight-for-age (Z-score)	-0.38 (1.02)	-0.18 (0.97)	-0.10 (0.97)	< 0.001
FEV_1_ (Z-score) [mean (SD)]**	-0.01 (0.29)	-0.02 (0.30)	-0.05 (0.30)	< 0.001
FVC (Z-score) [mean (SD)]**	0.00 (0.32)	0.01 (0.33)	-0.01 (0.33)	0.012
O_3_ ^§^ ppb	67.0 (11.4)	67.1 (10.5)	69.90 (12.5)	< 0.001

*Measured in phase 4 only

**Z-score was calculated with equation published by Martínez-Briseño et al. (13)

^§^Previous 6 months of the daily O_3_ 8-hour mean (parts per billion [ppb] from 10 a.m. to 6 p.m.).

Children of families with higher parental education in general were taller for age, had higher crude spirometric variables and, during the 4th evaluation, also had lower sitting height/standing height. Similarly, poorer children had smaller body and lung size, but in addition, smaller legs in relation to trunk size (Table 4).

Mixed regression models predicting log FEV_1_ (mL) and log FVC (mL) showed that the higher the monthly family income, the higher the lung function (models 1‒4). Parents’ schooling also was associated with higher lung function (model 1). When monthly income increased by 10%, FEV_1_ increased 0.008‒0.03% and FVC, 0.02‒0.04% (models 1‒4). However, only the co-efficient in model 1 was statistically significant for both FEV_1_ and FVC. A 1-year increase in parents’ schooling was associated with an increase of 0.37% for FEV_1_ and 0.41% for FVC (Table 5). Utilizing model 1 of Table 5 and average income ($300.40 USD), schooling (11.5 years), FEV_1_ (2,322 mL), and FVC (2,605 mL), an increment of $100 USD in monthly income increased FEV_1_ by 1.98 mL (0.09%) and FVC by 2.88 mL (0.11%), whereas a 1-year increment in parents’ schooling increased FEV_1_ by 8.5 mL (0.37%) and FVC by 10.6 mL (0.41%).

**Table 5:** Longitudinal models for spirometric variables and Socioeconomic status (SES), both genders taken together.

Variables	(1)	(2)	(3)	(4)
**Ln FEV1 (mL)**				
Ln (Monthly family income) ^¶^	0.00296*	0.00127	0.00126	0.000725
Parents’ schooling (years)	0.00366***	-0.001	-0.00101	-0.000649
Gender	0.0443***	0.0480***	0.0481***	0.0486***
Age (years)	0.176***	-0.00906	-0.00791	-0.00385
Age^2^ (years^2^)	-0.00220***	0.00198***	0.00193***	0.00166***
Height (cm)		0.0128***	0.0127***	0.0128***
Weight (kg)		0.00396***	0.00396***	0.00392***
Secondhand smoke			-0.00169	-0.00239**
O_3_ ^δ^ ppb				-0.00102***
Constant	5.961***	5.591***	5.587***	5.634***
SD (residuals)	0.0786	0.0694	0.0759	0.0754
Observations	11,954	11,954	11,950	11,950
AIC^§^	-19753.24	-23462.42	-23452.74	-23588.63
**Ln FVC (mL)**				
Ln (Monthly family income)	0.00379**	0.00215	0.00212	0.00175
Parents’ schooling (years)	0.00406***	-0.000441	-0.000452	-0.00023
Gender	0.0681***	0.0735***	0.0737***	0.0740***
Age (years)	0.153***	-0.0214***	-0.0187***	-0.0163***
Age^2^ (years^2^)	-0.00152***	0.00232***	0.00220***	0.00204***
Height (cm)		0.0113***	0.0113***	0.0113***
Weight (kg)		0.00513***	0.00513***	0.00511***
Secondhand smoke			-0.00428***	-0.00473***
O_3_ ^δ^ ppb				-0.000619***
Constant	6.226***	5.941***	5.934***	5.962***
SD (residuals)	0.0786	0.0694	0.0694	0.0692
Observations	11,961	11,961	11,957	11,957
AIC^§^	-21390.43	-25267.34	-25270	-25327.86

^¶^Natural logarithm of income in 2002 U.S. dollars (USD)

^δ^Previous 6 months of the daily O_3_ 8-hour mean (parts per billion [ppb] 10 a.m. to 6 p.m.)

^§^AIC: Akaike information criterion.

***p <0.01

**p <0.05

*p <0.1.

If anthropometry is added to the model (model 2, Table 5), the impact of SES on lung function became non-significant. Secondhand smoke and ozone levels also exerted adverse effects on lung function, but had no additional impact on the relationship between SES and lung function (model 4). Higher ozone levels were observed in areas with students with higher SES.

Mixed regression models by gender or including healthy and non-healthy children were also performed, observing co-efficients for SES variables were similar to those obtained by models with healthy children and including both genders (S4–S7 Tables). Finally, cross-sectional models obtained from the first observation of each individual were compared with the more adequate longitudinal mixed-regression models illustrated in Table 5, observing similar effects for parents’ schooling, but not for monthly family income compared with longitudinal models. Furthermore, interaction between SES variables and ozone were not statistically significant. Longitudinal models had a lower Akaike information criterion (AIC) than cross-sectional models (S8 Table), again showing better fit of the observations.

S9 Table shows the effect of SES over spirometric variables expressed as Z-score and confirmed the positive effect of monthly family income on FEV_1_ (P<0.1) and FVC (P<0.01). However parents’ schooling co-efficient was not statistically significant.

**Fig 1:**
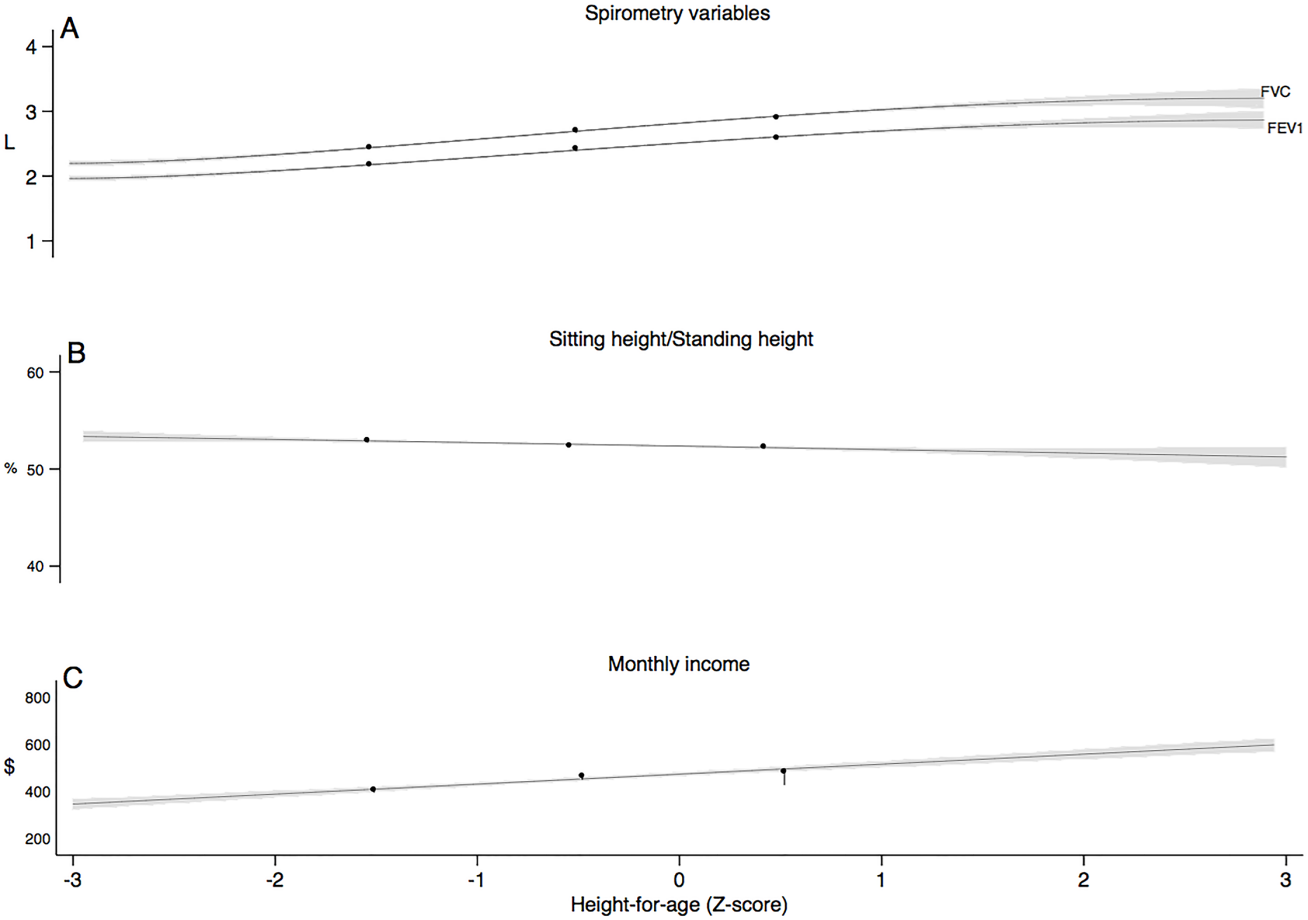
Relationship between height-for-age Z-score and spirometric, sitting height/standing height, and income. (A) Lung function (FVC and FEV_1_), (B) sitting height/standing height, and (C) monthly family income as a function of height for age as Z-score (horizontal axis). Line and shadows represent linear or quadratic regression and 95% Confidence intervals (95% CI) of the regression models. Symbols are means of terciles for height-for-age as described in the text, showing good fit of the models.

**Fig 2:**
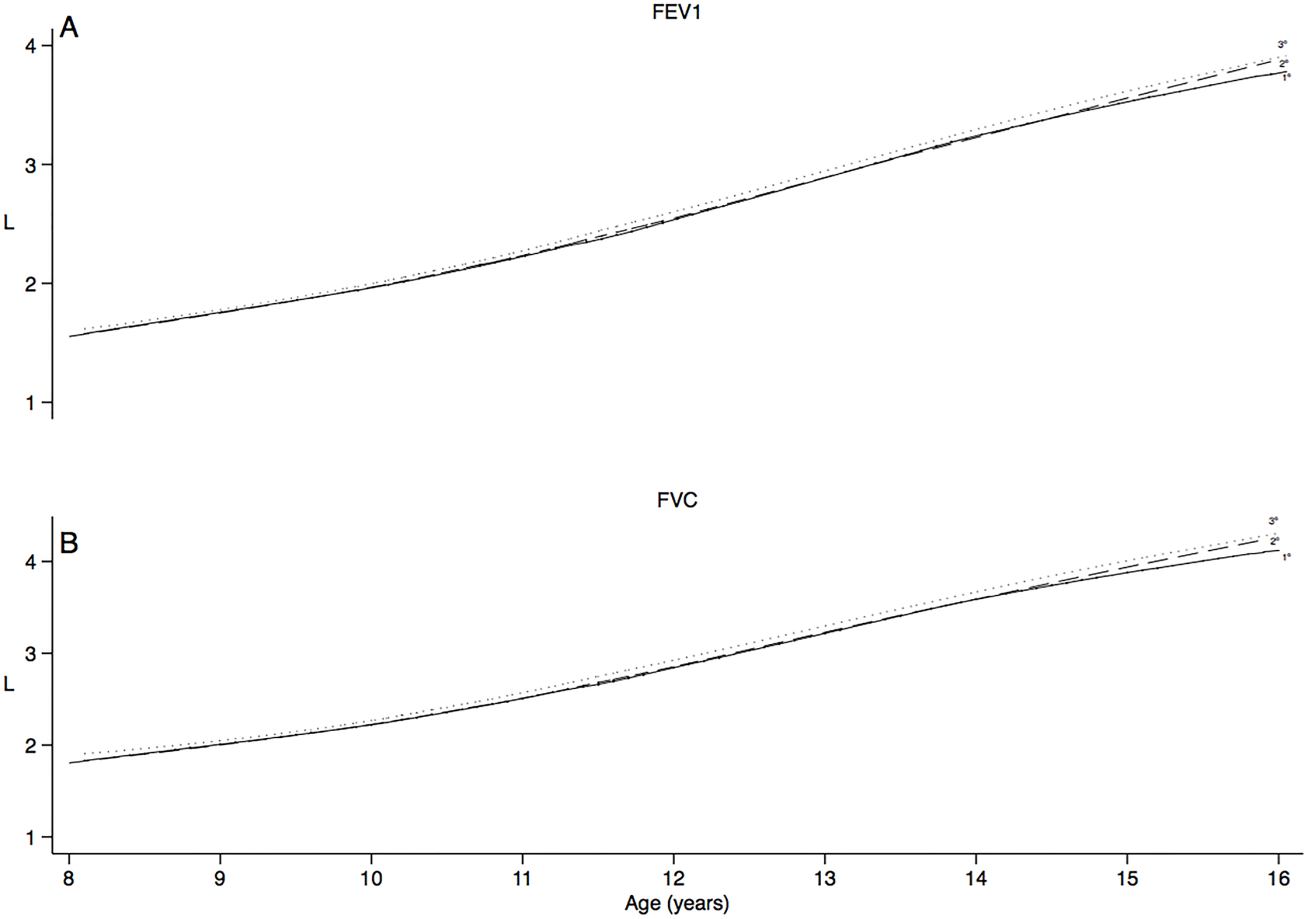
Forced expiratory volume at 1 sec (FEV_1_) and Forced vital capacity (FVC) expected as a function of age by tertiles of monthly family income. (A) FEV_1_ and (B) FVC as a function of age (horizontal axis). Lung function increases with growth and development in alinear fashion vs. age, but also varies with Socioeconomic status (SES) (shown as terciles in different lines), especially at the end of the observation.

Fig 1 depicts spirometric variables (L), sitting height/standing height ratio (%, from the 4th evaluation), and monthly family income (2009 USD) of the cohort vs. height-for-age Z-score. As height-for-age increases, spirometric variables and income also increase, but sitting height/standing height decreases. Fig 2 shows crude FEV_1_ and FVC in L vs. age. Children in the uppermost tertile of monthly family income had a slight higher lung function over time than children in the first two tertiles.

## Discussion

Our study shows, in a cohort of school age children residing 2,240 m above sea level, a lower FEV_1_ and FVC in families with lower monthly income or lower parents’ schooling. Socioeconomic disparities are associated with differences in children’s lung function. The majority of studies to date, many of these cross-sectional, demonstrate a positive correlation between SES (income, education, occupation, and others, or a combination of these in an index) and lung function [15], as our results describe.

The detailed mechanisms of why SES adversely impacts lung function are unknown, but are likely multifactorial and beyond the common explanation of poor nutrition [3]. SES adversely impacts growth and development; therefore, several anthropometric measurements, such as height-for-age and sitting height/standing height [6, 16–19], are important predictors of body size and overall lung size and function, and are likely key intermediate variables between SES and lung function. In fact, in our study, the impact of SES on lung function disappeared on adjusting regression models by anthropometry, observing an expected adverse effect on lung function and air pollutants (exemplified by ozone levels) and passive smoking, supporting the idea that the majority of the effect of SES on lung function is associated with changes in height and sitting height/standing height. In other words, poverty leads to smaller body size, lung size, and crude lung function, but not to height- and age-adjusted lung function. Changes in height-adjusted lung function, for example, when function is expressed as percentage predicted by gender, age, and height, or as Z- score of gender-, age-, and height-predicted values, were mild and inconsistent in different tertiles of income or education.

Compared with children in developed countries, including Mexican-American children residing close to sea level, gender-, age-, and height-adjusted lung function of children from Mexico City have been higher [14] suggesting that an adverse environmental impact on height is more marked than on the lung, especially in individuals residing at moderate or high altitudes. However, crude lung function in Mexican-American children is clearly higher than in children of the same age residing in Mexico City, as also shown in the groups with higher SES within Mexico City; improving nutrition during growth and development could raise the maximal lung function attained and decrease the risk of adult-age lung diseases such as Chronic obstructive pulmonary disease (COPD) [20, 21]. Another source of the adverse impact of low SES on lung function derives from air pollution, which tends to be worse in poorer areas. For example, solid fuel use, very much a pollutant, is linked with poverty; however, in our study, the use of solid fuels is very uncommon and ozone levels were higher in more highly advantaged areas. In addition, previous studies have found a differential impact of air pollutants depending on SES, but not consistently [22]. For example benzene, NO_2_, and NOx [21], and Ozone can be modified by maternal educational level or SES, respectively, but particulate matter and NO_2_ have a small decrease in lung function, even when SES was taken into account in the models. [23].

Several study limitations should be acknowledged. Income has disadvantages on measuring SES, because it is often under-reported, although income may change over time, reflecting better modifications in family economy during follow-up. Schooling, on the other hand, does not change over time, even with changes in income or poverty, but is able to be measured with greater reliability. More complex indicators of SES have been proposed, with composite indices including existent in-home appliances, but these are not necessarily better than traditional family income or education.

Longitudinal follow-up of the cohort ended when the children were about 15 years of age, during adolescence, but before final height and lung function were reached. In addition, as can be observed in Tables 1 and 2, follow-up decreased during secondary school due to the children’s dropping out of school or changing to a different school, reducing information at the beginning of adolescence. We also lacked contributions of genetic ancestry, because biological samples were not obtained and questions on race or ethnic origin are usually avoided in Mexico, as the majority of the population is considered Mexican mestizo and differs from the so-called Hispanic population in the U.S., with a variety of contributions of African ancestry depending on country-of-origin. On the other hand, the study includes a considerable number of spirometric observations conducted with good quality control, as well as measurements of air pollution and a heterogeneous socioeconomic status of the individuals studied, allowing for relevant analysis.

## Conclusions

Lower socioeconomic status, estimated as reported income or parents’ education, is associated with a reduction in lung function and height-for-age and higher sitting height/standing height. According to our data, the majority of the impact of SES on lung function was accomplished by means of a reduction in height. Further studies are needed in order to explore the potential factors underling this association and the impact of residing at a moderate altitude.

## Supporting Information

S1 Table Main characteristics of boys studied (means and Standard deviation [SD]). Population characteristics.
(DOC)

S2 Table Main characteristics of girls studied (means and Standard deviation [SD]). Population characteristics.
(DOC)

S3 Table Anthropometric and spirometric variables by tertiles of monthly family income. Population characteristics.
(DOC)

S4 Table Longitudinal models for Socioeconomic status (SES) and lung function in girls. (DOC)

S5 Table Longitudinal models for Socioeconomic status (SES) and lung function in boys. (DOC)

S6 Table Longitudinal models for Socioeconomic status (SES) and lung function including unhealthy girls. (DOC)

S7 Table Longitudinal models for Socioeconomic status (SES) and lung function including unhealthy boys. (DOC)

S8 Table Cross-sectional models for spirometric variables and Socioeconomic status (SES), both genders. (DOC)

S9 Table Longitudinal models for spirometric variables expressed as Z-score and Socioeconomic status (SES), both genders taken together. (DOC)

S1 Dataset Compressed Dataset in Excel (XLS) format. (XLS)
