# Supplementary material for: Socioeconomic Status and Longitudinal Lung Function of Healthy Mexican Children
Source: PLoS One. 2015 Sep 17;10(9):e0136935. doi: 10.1371/journal.pone.0136935 (PMC4574937; doi:10.1371/journal.pone.0136935)
Supplement: S1 Table — Population characteristics. (DOC) [file pone.0136935.s001.doc]

**S1 Table. Main characteristics of the boys studied (means and Standard deviation [SD])**

|  |  | Age | Weight | Height | BMI | FEV_1_ | FVC | PEF | FEV_1_/FVC | Height-for-age | Weight-for-age |
| --- | --- | --- | --- | --- | --- | --- | --- | --- | --- | --- | --- |
| Phase | *N* | (years) | (kg) | (cm) | (kg/m2) | (L) | (L) | (L/s) | (%) | (Z-score) | (Z-score) |
| 1 | 676 | 9.3 (0.7) | 28.2 (4.9) | 130.4 (6.1) | 16.5 (1.9) | 1.85 (0.31) | 2.18 (0.37) | 4.02 (0.87) | 85.3 (7.9) | –0.73 (0.91) | –0.48 (1.19) |
| 2 | 718 | 9.7 (0.8) | 29.8 (5.3) | 133.0 (6.4) | 16.8 (2.0) | 1.95 (0.32) | 2.24 (0.37) | 4.55 (0.81) | 87.3 (6.4) | –0.67 (0.93) | –0.42 (1.03) |
| 3 | 853 | 10.2 (0.8) | 32.0 (6.1) | 136.1 (6.6) | 17.2 (2.3) | 2.06 (0.33) | 2.35 (0.38) | 4.95 (0.87) | 87.8 (5.7) | –0.56 (0.91) | –0.34 (1.07) |
| 4 | 776 | 10.7 (0.8) | 34.3 (6.8) | 138.9 (7.3) | 17.6 (2.3) | 2.19 (0.38) | 2.49 (0.42) | 5.24 (0.93) | 88.2 (5.6) | –0.51 (0.93) | –0.27 (1.03) |
| 5 | 657 | 11.2 (0.8) | 36.4 (7.3) | 142.1 (7.7) | 17.9 (2.4) | 2.25 (0.41) | 2.57 (0.47) | 5.36 (1.01) | 87.9 (5.8) | –0.42 (0.94) | –0.26 (1.02) |
| 6 | 808 | 11.7 (0.8) | 38.6 (8.1) | 144.8 (8.2) | 18.3 (2.5) | 2.50 (0.49) | 2.83 (0.54) | 6.00 (1.12) | 88.3 (5.7) | –0.45 (0.98) | –0.28 (1.04) |
| 7 | 812 | 12.2 (0.8) | 41.0 (8.3) | 148.1 (8.2) | 18.5 (2.5) | 2.64 (0.54) | 3.00 (0.59) | 6.28 (1.23) | 88.1 (6.0) | –0.34 (1.00) | –0.23 (1.02) |
| 8 | 322 | 13.1 (0.6) | 46.2 (9.0) | 154.0 (8.0) | 19.3 (2.6) | 3.07 (0.60) | 3.43 (0.64) | 7.18 (1.42) | 89.5 (5.6) | –0.41 (0.93) | –0.20 (1.09) |
| 9 | 282 | 13.6 (0.6) | 48.3 (9.0) | 157.3 (7.9) | 19.4 (2.5) | 3.27 (0.65) | 3.68 (0.70) | 7.63 (1.54) | 89.0 (5.9) | –0.44 (0.92) | –0.22 (0.97) |
| 10 | 280 | 14.1 (0.6) | 50.7 (8.9) | 160.0 (7.5) | 19.7 (2.6) | 3.50 (0.64) | 3.91 (0.70) | 8.08 (1.61) | 89.6 (5.9) | –0.50 (0.87) | –0.21 (0.95) |
| 11 | 231 | 14.6 (0.5) | 53.5 (8.8) | 162.9 (6.9) | 20.1 (2.5) | 3.95 (0.69) | 4.40 (0.76) | 9.02 (1.74) | 89.8 (6.0) | –0.54 (0.83) | –0.17 (0.92) |
| 12 | 240 | 15.0 (0.6) | 55.1 (9.2) | 164.5 (6.9) | 20.3 (2.8) | 3.86 (0.64) | 4.28 (0.72) | 8.98 (1.56) | 90.3 (6.3) | –0.61 (0.84) | –0.23 (0.97) |

SD = Standard deviation; Phase = Study phase, evaluations twice annually during spring and autumn; BMI = Body mass index; FEV_1_ = Forced expiratory volume; FVC = Forced vital capacity; FEV_1_/FVC = ratio of FEV_1_ and FVC, PEF = Peak expiratory flow. Mean FEV_1_, FVC, and PEF in the last evaluation are slightly lower than in the 11th evaluation, but not those of all individuals evaluated were the same.
