# Supplementary material for: Socioeconomic Status and Longitudinal Lung Function of Healthy Mexican Children
Source: PLoS One. 2015 Sep 17;10(9):e0136935. doi: 10.1371/journal.pone.0136935 (PMC4574937; doi:10.1371/journal.pone.0136935)
Supplement: S2 Table — Population characteristics. (DOC) [file pone.0136935.s002.doc]

**S2 Table. Main characteristics of girls studied (means and Standard deviation [SD])**

|  |  | Age | Weight | Height | BMI | FEV_1_ | FVC | PEF | FEV_1_/FVC | Height-for-age | Weight-for-age |
| --- | --- | --- | --- | --- | --- | --- | --- | --- | --- | --- | --- |
| Phase | *N* | (years) | (kg) | (cm) | (kg/m2) | (L) | (L) | (L/s) | (%) | (Z-score) | (Z-score) |
| 1 | 776 | 9.1 (0.7) | 28.4 (5.4) | 130.1 (6.2) | 16.7 (2.3) | 1.72 (0.29) | 1.98 (0.33) | 3.81 (0.88) | 87.1 (8.3) | –0.61 (0.93) | –0.42 (1.07) |
| 2 | 795 | 9.6 (0.7) | 30.0 (5.6) | 132.9 (6.3) | 16.9 (2.3) | 1.81 (0.29) | 2.04 (0.33) | 4.29 (0.87) | 88.9 (6.2) | –0.51 (0.89) | –0.38 (0.96) |
| 3 | 949 | 10.1 (0.7) | 32.6 (6.4) | 136.6 (6.7) | 17.3 (2.4) | 1.96 (0.33) | 2.19 (0.36) | 4.80 (0.93) | 89.7 (5.3) | –0.35 (0.93) | –0.30 (0.97) |
| 4 | 895 | 10.6 (0.7) | 35.2 (6.9) | 139.9 (7.3) | 17.9 (2.5) | 2.11 (0.35) | 2.33 (0.39) | 5.18 (0.96) | 90.5 (5.1) | –0.28 (0.96) | –0.21 (0.96) |
| 5 | 748 | 11.1 (0.7) | 37.4 (7.3) | 142.7 (7.1) | 18.2 (2.6) | 2.18 (0.38) | 2.41 (0.42) | 5.34 (1.00) | 90.3 (5.7) | –0.32 (0.96) | –0.20 (0.95) |
| 6 | 892 | 11.6 (0.7) | 40.2 (7.6) | 145.7 (6.9) | 18.9 (2.7) | 2.44 (0.41) | 2.69 (0.45) | 6.05 (1.09) | 90.8 (5.6) | –0.35 (0.93) | –0.10 (0.92) |
| 7 | 912 | 12.1 (0.7) | 42.7 (8.0) | 148.7 (6.6) | 19.2 (2.9) | 2.57 (0.42) | 2.84 (0.46) | 6.36 (1.08) | 90.7 (5.5) | –0.36 (0.92) | –0.05 (0.92) |
| 8 | 351 | 13.0 (0.6) | 47.8 (8.3) | 152.6 (5.9) | 20.5 (3.0) | 2.93 (0.43) | 3.19 (0.47) | 7.12 (1.15) | 92.1 (5.1) | –0.64 (0.86) | 0.05 (0.93) |
| 9 | 327 | 13.5 (0.6) | 49.1 (7.5) | 154.0 (5.6) | 20.7 (2.7) | 3.02 (0.43) | 3.31 (0.47) | 7.27 (1.18) | 91.4 (5.3) | –0.73 (0.84) | 0.03 (0.81) |
| 10 | 317 | 14.0 (0.6) | 50.7 (7.5) | 154.9 (5.5) | 21.1 (2.8) | 3.09 (0.44) | 3.40 (0.49) | 7.40 (1.19) | 91.3 (5.4) | –0.78 (0.84) | 0.04 (0.79) |
| 11 | 269 | 14.5 (0.6) | 51.9 (6.9) | 155.7 (5.3) | 21.4 (2.5) | 3.30 (0.43) | 3.63 (0.48) | 7.81 (1.24) | 91.0 (5.7) | –0.83 (0.82) | 0.03 (0.71) |
| 12 | 279 | 14.9 (0.6) | 52.0 (7.1) | 155.8 (5.3) | 21.4 (2.7) | 3.12 (0.39) | 3.43 (0.44) | 7.62 (1.18) | 91.4 (5.7) | –0.89 (0.83) | –0.08 (0.80) |

SD = Standard deviation; Phase = Study phase, evaluations twice annually during spring and autumn; BMI = Body mass index; FEV_1_ = Forced expiratory volume in 1 sec; FVC = Forced vital capacity; FEV_1_/FVC = Ratio of FEV_1_ and FVC; PEF = Peak expiratory flow. Mean FEV_1_, FVC, and PEF in the last evaluation are slightly lower than in the 11th evaluation, but not all individuals evaluated were the same.
