# Supplementary material for: Socioeconomic Status and Longitudinal Lung Function of Healthy Mexican Children
Source: PLoS One. 2015 Sep 17;10(9):e0136935. doi: 10.1371/journal.pone.0136935 (PMC4574937; doi:10.1371/journal.pone.0136935)
Supplement: S3 Table — Population characteristics. (DOC) [file pone.0136935.s003.doc]

**S3 Table. Anthropometric and spirometric variables by tertiles of family monthly income (2002 USD)**

| Variable | 1 (min 47, max 228) | 2 (min 266, max 531) | 3 (min 559, max 1858) | P Value |
| --- | --- | --- | --- | --- |
| FEV_1_ (L) [Mean (SD)] | 2.52 (0.70) | 2.36 (.64) | 2.44 (0.73) | <0.001 |
| FVC (L) [Means (SD)] | 2.82 (0.76) | 2.64 (.70) | 2.74 (0.79) | <0.001 |
| FEV_1_/FVC (%) [Mean (SD)] | 90.0 (6.0) | 89.4 (6.0) | 89.0 (6.0) | <0.001 |
| Age (Years [Mean (SD)] | 11.8 (1.7) | 11.3 (1.6) | 11.4 (1.9) | <0.001 |
| Standing height (cm) [Mean (SD)] | 145 (11.2) | 143 (10.7) | 144 (12.0) | <0.001 |
| Sitting height /Standing height (%) [Mean (SD)]* | 53.0 (2.3) | 52.7 (2.4) | 52.5 (2.3) | <0.001 |
| Weight (Kg) [Mean (SD)] | 39.9 (10.6) | 37.8 (10.0) | 38.7 (10.7) | <0.001 |
| BMI (Kg/m^2^) [Mean (SD)] | 18.7 (2.9) | 18.3 (2.8) | 18.3 (2.8) | <0.001 |
| BMI-for-age (Z-score) | 0.08 (0.96) | 0.04 (1.36) | 0.04 (1.12) | 0.192 |
| Height-for-age (Z-score) | -0.61 (0.94) | -0.47 (0.92) | -0.35 (0.91) | <0.001 |
| Weight-for-age (Z-score) | -0.27 (0.97) | -0.23 (1.0) | -0.16 (0.99) | <0.001 |
| FEV_1_ (Z-score) [Mean(SD)]** | -0.01 (0.32) | -0.03 (0.29) | -0.04 (0.30) | <0.001 |
| FVC (Z-score) [Mean(SD)]** | 0.01 (0.35) | -0.01 (0.32) | 0.00 (0.33) | 0.0119 |
| O_3_^§^ | 66.6 (11.1) | 67.6 (10.7) | 69.50 (12.8) | <0.001 |

*Measured in phase 4 only

** Z-score was calculated with equation published by Martínez-Briseño et al. (14)

^§^ Previous 6 months of the daily O_3_ 8-hour mean (parts per billion [ppb] from 10 a.m. to 6 p.m.).
