# Supplementary material for: Socioeconomic Status and Longitudinal Lung Function of Healthy Mexican Children
Source: PLoS One. 2015 Sep 17;10(9):e0136935. doi: 10.1371/journal.pone.0136935 (PMC4574937; doi:10.1371/journal.pone.0136935)
Supplement: S4 Table — (DOC) [file pone.0136935.s004.doc]

**S4 Table. Longitudinal models for Socioeconomic status (SES) and lung function in girls**

| Variables | (1) | (2) | (3) | (4) |
| --- | --- | --- | --- | --- |
| **Ln FEV1 (mL)** |  |  |  |  |
| Ln(Monthly family income) ^¶^ | 0.00132 | 0.00173 | 0.00171 | 0.00109 |
| Parents' schooling (years) | 0.00483*** | -9.42E-05 | -9.68E-05 | 9.19E-05 |
| Age (years) | 0.324*** | 0.0753*** | 0.0771*** | 0.0779*** |
| Age^2^ (years^2^) | -0.00875*** | -0.00122*** | -0.00129*** | -0.00143*** |
| Height (cm) |  | 0.0110*** | 0.0110*** | 0.0111*** |
| Weight (Kg) |  | 0.00423*** | 0.00421*** | 0.00420*** |
| Secondhand smoke |  |  | -0.00263 | -0.00330** |
| O_3_^δ^ ppb |  |  |  | -0.000877*** |
| Constant | 5.135*** | 5.285*** | 5.279*** | 5.327*** |
| SD (residual) | 0.0809 | 0.0764 | 0.0764 | 0.0761 |
| Observations | 6,446 | 6,446 | 6,442 | 6,442 |
| AIC^§^ | -11348.21 | -12590.08 | -12581.67 | -12632.74 |
|  |  |  |  |  |
| **Ln FVC (mL)** |  |  |  |  |
| Ln(Monthly family income) ^¶^ | 0.00357* | 0.00355* | 0.00348* | 0.00309 |
| Parents' schooling (years) | 0.00505*** | 0.000197 | 0.000194 | 0.000309 |
| Age (years) | 0.282*** | 0.0402*** | 0.0442*** | 0.0446*** |
| Age^2^ (years^2^) | -0.00723*** | -4.81E-05 | -0.000212 | -0.000291 |
| Height (cm) |  | 0.00985*** | 0.00981*** | 0.00992*** |
| Weight (Kg) |  | 0.00562*** | 0.00559*** | 0.00558*** |
| Secondhand smoke |  |  | -0.00649*** | -0.00690*** |
| O_3_^δ^ ppb |  |  |  | -0.000524*** |
| Constant | 5.507*** | 5.729*** | 5.718*** | 5.746*** |
| SD (residual) | 0.0766 | 0.0714 | 0.0714 | 0.0713 |
| Observations | 6,455 | 6,455 | 6,451 | 6,451 |
| AIC^§^ | -11946 | -13041.95 | -13348.49 | -13368.01 |

^¶^ Natural logarithm of income in US Dollars of 2002; ^δ^Previous 6 months of the daily O_3_ 8-hour mean (parts per billion [ppb] 10 A.M. to 6 P.M.); ^§^AIC: Akaike information criterion; ***p <0.01; **p <0.05; *p <0.1.
