# Supplementary material for: Socioeconomic Status and Longitudinal Lung Function of Healthy Mexican Children
Source: PLoS One. 2015 Sep 17;10(9):e0136935. doi: 10.1371/journal.pone.0136935 (PMC4574937; doi:10.1371/journal.pone.0136935)
Supplement: S5 Table — (DOC) [file pone.0136935.s005.doc]

**S5 Table. Longitudinal models for Socioeconomic status (SES) and lung function in boys**

| Variables | (1) | (2) | (3) | (4) |
| --- | --- | --- | --- | --- |
| **Ln FEV_1_ (mL)** |  |  |  |  |
| Ln(Monthly family income) ^¶^ | 0.000236 | -0.000287 | -0.000289 | -0.000653 |
| Parents' schooling (years) | 0.00296** | -0.00135 | -0.00135 | -0.000831 |
| Age (years) | 0.00515 | -0.0792*** | -0.0790*** | -0.0728*** |
| Age^2^ (years^2^) | 0.00527*** | 0.00475*** | 0.00474*** | 0.00438*** |
| Height (cm) |  | 0.0137*** | 0.0137*** | 0.0137*** |
| Weight (Kg) |  | 0.00325*** | 0.00326*** | 0.00320*** |
| Secondhand smoke |  |  | -0.000235 | -0.000947 |
| O_3_^δ^ ppb |  |  |  | -0.00113*** |
| Constant | 6.983*** | 5.981*** | 5.981*** | 6.025*** |
| SD (residual) | 0.0824 | 0.0738 | 0.0739 | 0.0732 |
| Observations | 5,508 | 5,508 | 5,508 | 5,508 |
| AIC^§^ | -9319.8 | -11021.1 | -11019.12 | -11101.29 |
|  |  |  |  |  |
| **Ln FVC (mL)** |  |  |  |  |
| Ln(Monthly family income) ^¶^ | -1.87E-05 | -0.00028 | -0.000296 | -0.000585 |
| Parents' schooling (Years) | 0.00355** | -0.00055 | -0.000556 | -0.000234 |
| Age (years) | 0.00554 | -0.0717*** | -0.0708*** | -0.0671*** |
| Age^2^ (years^2^) | 0.00498*** | 0.00438*** | 0.00434*** | 0.00412*** |
| Height (cm) |  | 0.0120*** | 0.0120*** | 0.0120*** |
| Weight (Kg) |  | 0.00425*** | 0.00426*** | 0.00422*** |
| Secondhand smoke |  |  | -0.00138 | -0.00184 |
| O_3_^δ^ ppb |  |  |  | -0.000691*** |
| Constant | 7.142*** | 6.264*** | 6.262*** | 6.289*** |
| SD (residual) | 0.074 | 0.0661 | 0.0661 | 0.0658 |
| Observations | 5,506 | 5,506 | 5,506 | 5,506 |
| AIC^§^ | -10296.11 | -12036.03 | -12034.79 | -12071.02 |

^¶^ Natural logarithm of income in US Dollars of 2002; ^δ^Previous 6 months of the daily O_3_ 8-hour mean (parts per billion [ppb] 10 A.M. to 6 P.M.); ^§^AIC: Akaike information criterion; ***p <0.01; **p <0.05; *p <0.1.
