# Supplementary material for: Socioeconomic Status and Longitudinal Lung Function of Healthy Mexican Children
Source: PLoS One. 2015 Sep 17;10(9):e0136935. doi: 10.1371/journal.pone.0136935 (PMC4574937; doi:10.1371/journal.pone.0136935)
Supplement: S8 Table — (DOC) [file pone.0136935.s008.doc]

**S8 Table. Cross-sectional models for spirometric variables and Socioeconomic status (SES), both genders taken together**

| Variables | (1) | (2) | (3) | (4) |
| --- | --- | --- | --- | --- |
| **Ln FEV_1_ (mL)** |  |  |  |  |
| Ln(Monthly family income) ^¶^ | -0.00374 | -0.00896** | -0.00901** | -0.00816** |
| Parents' schooling (years) | 0.00517*** | 0.000334 | 0.000379 | 0.000508 |
| Sex | 0.0594*** | 0.0661*** | 0.0663*** | 0.0663*** |
| Age (years) | -0.0174 | -0.0713* | -0.0743** | -0.0789** |
| Age^2^ (years^2^) | 0.00632*** | 0.00420** | 0.00432** | 0.00449** |
| Height (cm) |  | 0.0138*** | 0.0138*** | 0.0138*** |
| Weight (Kg) |  | 0.00446*** | 0.00446*** | 0.00447*** |
| Secondhand smoke |  |  | 0.00371 | 0.00298 |
| O_3_^δ^ ppb |  |  |  | -0.000483* |
| Constant | 7.043*** | 5.863*** | 5.880*** | 5.943*** |
| Observations | 1,685 | 1,685 | 1,681 | 1,681 |
| R^2^ | 0.371 | 0.606 | 0.606 | 0.607 |
| AIC^§^ | -1214.38 | -1999.06 | -1989.574 | -1990.708 |
|  |  |  |  |  |
| **Ln FVC (mL)** |  |  |  |  |
| Ln(Monthly family income) ^¶^ | -0.0012 | -0.00593* | -0.00586 | -0.00613* |
| Parents' schooling (years) | 0.00512*** | 0.000441 | 0.00045 | 0.000409 |
| Sex | 0.0790*** | 0.0856*** | 0.0855*** | 0.0855*** |
| Age (years) | -0.0405 | -0.0772** | -0.0760** | -0.0746** |
| Age^2^ (years^2^) | 0.00719*** | 0.00439*** | 0.00434*** | 0.00429*** |
| Height (cm) |  | 0.0123*** | 0.0123*** | 0.0123*** |
| Weight (Kg) |  | 0.00562*** | 0.00563*** | 0.00562*** |
| Secondhand smoke |  |  | -0.00184 | -0.00161 |
| O_3_^δ^ ppb |  |  |  | 0.000155 |
| Constant | 7.301*** | 6.182*** | 6.175*** | 6.154*** |
| Observations | 1,686 | 1,686 | 1,682 | 1,682 |
| R^2^ | 0.39 | 0.627 | 0.627 | 0.627 |
| AIC^§^ | -1392.11 | -2220.06 | -2209.7 | -2208.066 |

^¶^ Natural logarithm of income in US Dollars of 2002; ^δ^Previous 6 months of the daily O_3_ 8-hour mean (parts per billion [ppb] 10 A.M. to 6 P.M.); ^§^AIC: Akaike information criterion; ***p <0.01; **p <0.05; *p <0.1.
