# Supplementary material for: Socioeconomic Status and Longitudinal Lung Function of Healthy Mexican Children
Source: PLoS One. 2015 Sep 17;10(9):e0136935. doi: 10.1371/journal.pone.0136935 (PMC4574937; doi:10.1371/journal.pone.0136935)
Supplement: S9 Table — (DOC) [file pone.0136935.s009.doc]

**S9 Table. Longitudinal models for spirometric variables expressed as Z-score and Socioeconomic status (SES), both genders taken together**

| Variables | (1) | (2) | (3) |
| --- | --- | --- | --- |
| **FEV_1_ (Z-score)** |  |  |  |
| Ln(Monthly family income) ^¶^ | 0.0398** | 0.0385** | 0.0318* |
| Parents' schooling (years) | -0.0149* | -0.0108 | -0.00883 |
| Gender | 0.0582 | 0.0625 | 0.0654 |
| Secondhand smoke | -0.0389** | -0.0393** | -0.0434*** |
| O_3_^δ^ ppb |  | -0.0128*** | -0.0137*** |
| Height (cm) |  |  | -0.00309*** |
| Constant | -0.013 | 0.821*** | 1.341*** |
| SD(residual) | 0.99 | 0.99 | 0.99 |
| Observations | 11,950 | 11,950 | 11,950 |
| AIC^§^ | 38014.91 | 37872.2 | 37866.77 |
|  |  |  |  |
| **FVC (Z-score)** |  |  |  |
| Ln(Monthly family income) ^¶^ | 0.0567*** | 0.0552*** | 0.0526*** |
| Parents' schooling (years) | -0.00763 | -0.00458 | -0.00384 |
| Gender | -0.0256 | -0.0225 | -0.0215 |
| Secondhand smoke | -0.0829*** | -0.0833*** | -0.0849*** |
| O_3_^δ^ ppb |  | -0.00935*** | -0.00967*** |
| Height (cm) |  |  | -0.00114 |
| Constant | -0.242* | 0.369** | 0.561** |
| SD(residual) | 1.00 | 1.00 | 1.00 |
| Observations | 11,957 | 11,957 | 11,957 |
| AIC^§^ | 38531.02 | 38458.05 | 38459.09 |

^¶^Natural logarithm of income in 2002 U.S. dollars (USD); ^δ^Previous 6 months of the daily O_3_ 8-hour mean (parts per billion [ppb] 10 a.m. to 6 p.m.); ^§^AIC: Akaike information criterion. ****p* <0.01; ***p* <0.05; **p* <0.1.
